# Evolutionary Mode and Functional Divergence of Vertebrate NMDA Receptor Subunit 2 Genes

**DOI:** 10.1371/journal.pone.0013342

**Published:** 2010-10-14

**Authors:** Huajing Teng, Wanshi Cai, LingLin Zhou, Jing Zhang, Qi Liu, Yongqing Wang, Wei Dai, Mei Zhao, Zhongsheng Sun

**Affiliations:** 1 Behavioral Genetics Center, Institute of Psychology, Chinese Academy of Science, Beijing, China; 2 Institute of Genomic Medicine, Wenzhou Medical College, Wenzhou, China; American Museum of Natural History, United States of America

## Abstract

**Background:**

Ionotropic glutamate receptors in the central nervous system play a major role in numerous brain functions including learning and memory in many vertebrate species. NR2 subunits have been regarded as rate-limiting molecules in controlling the optimal *N*-methyl-*D*-aspartate (NMDA) receptor's coincidence-detection property and subsequent learning and memory function across multi-species. However, its evolutionary mode among vertebrate species remains unclear.

**Results:**

With extensive analysis of phylogeny, exon structure, protein domain, paralogon and synteny, we demonstrated that two-round genome duplication generated quartet GRIN2 genes and the third-round fish-specific genome duplication generated extra copies of fish GRIN2 genes. In addition, in-depth investigation has enabled the identification of three novel genes, *GRIN2C_Gg*, *GRIN2D-1_Ol* and *GRIN2D-2_Tr* in the chicken, medaka and fugu genome, respectively. Furthermore, we showed functional divergence of NR2 genes mostly occurred at the first-round duplication, amino acid residues located at the N-terminal Lig_chan domain were responsible for type I functional divergence between these GRIN2 subfamilies and purifying selection has been the prominent natural pressure operating on these diversified GRIN2 genes.

**Conclusion and Significance:**

These findings provide intriguing subjects for testing the 2R and 3R hypothesis and we expect it could provide new insights into the underlying evolution mechanisms of cognition in vertebrate.

## Introduction

The neurotransmitter glutamate signals via ionotropic glutamate receptors (iGluRs) in the central nervous system play a major role in numerous brain functions including learning and memory, and show a remarkable level of conservation among vertebrate species [1], [2]. Understanding the evolutionary mode and tempo of these receptors would shed substantial novel insights into the underlying evolution mechanisms of cognition in vertebrate.

The *N*-methyl-*D*-aspartate (NMDA) subtype of glutamate receptors are the molecular switches for synaptic plasticity [3] and learning and memory [4], [5], [6], [7] in both invertebrates and vertebrates. Functional NMDA receptors are composed of NR1 and at least of one of the NR2 (A, B, C, and D), or NR3 (A and B) subunits [8], [9]. NR2 subunits not only provide glutamate-binding sites in the NMDA receptor complex [10], but also modify the channel properties [9], [11]. Furthermore, through binding to post-synaptic anchoring proteins, NR2 subunits can control cell surface expression and subcellular localization of the NMDA receptors [12], [13], [14]. Thus, NR2 subunits have been regarded as rate-limiting molecules in controlling the optimal NMDA receptor's coincidence-detection property and subsequent learning and memory function across multi-species [6]. Currently, four NR2 subunit genes (*GRIN2A*, *GRIN2B*, *GRIN2C* and *GRIN2D*) with distinct spatial and temporal expression patterns are identified in vertebrates, except for more counterparts in teleost fishes. In contrast, only a single NR2 orthologue has been detected in the invertebrate lineage. These observations suggest that the four tetrapod NR2 subunit genes might be obtained by gene duplication [15]. However, no further evidence has been obtained and the evolutionary mode of these NR2 subunit genes remains unclear.

Gene duplication has been considered as a powerful mechanism in evolution and whole-genome duplication has been recognized as a parsimonious evolutionary innovation [16], [17], [18]. Two rounds of genome duplications (2R hypothesis) occurred during the emergence of vertebrates have been proposed by Ohno [17]. The discovery of seven *Hox* gene clusters in zebrafish, which are almost twice as many as in human or mouse, has challenged the 2R hypothesis. A doubly conserved synteny between the chromosomes of the teleosts compared to those of human [19], [20], [21], [22] demonstrated that a fish-specific whole-genome duplication had indeed occurred after the divergence of ray-finned and lobe-finned fishes but before the teleost radiation (3R hypothesis) [23]. However, due to the deletion or translocation of genes or chromosomes and extensive chromosomal rearrangements, it complicates the deciphering of ancestral gene landscapes [24]. A single NR2 gene in invertebrate and multiple orthologues in vertebrate lineage provide intriguing subjects for testing the 2R and 3R hypothesis.

In this study, integrated analysis of phylogeny, exon structure, protein domain, paralogon and synteny were applied to elucidate the evolutionary relationships of vertebrate NR2 subunit genes and denominate additional NR2 subunit genes of some species in terms of evolution. In addition, type I functional divergence and natural selection were conducted to examine whether functional divergence has occurred following the gene duplication. With such in-depth investigation, we expected to provide more insights into the underlying evolution mechanisms of cognition in vertebrate and provide intriguing subjects for testing the 2R and 3R hypothesis.

## Results

### Phylogenetic analysis of NR2 subunit genes

Through extensive similarity-based searches, we identified 53 vertebrate NR2 subunit genes: 4 in human, mouse, zebra finch and chicken, 5 in anole lizard, 8 in each of the four teleost fishes (Table S1). Among them, 49 have been identified in Ensemble or NCBI databases. Two pseudo genes (*GRIN2D_Tg* and *GRIN2D_Gg*) were identified in zebra finch and chicken genome, and one of them (*GRIN2D_Gg*) has been reported by Hillier previously [25]. One novel *GRIN2C_Gg* gene was identified to reside in the chicken Chromosome 18. Due to the sequenced gap of this location, however, only 7 exons were obtained using GENSCAN prediction. Additionally, two novel genes, *GRIN2D-1_Ol* and *GRIN2D-2_Tr*, were identified to reside in the medaka Chromosome 11 and fugu scaffold_202, respectively.

**Figure 1:**
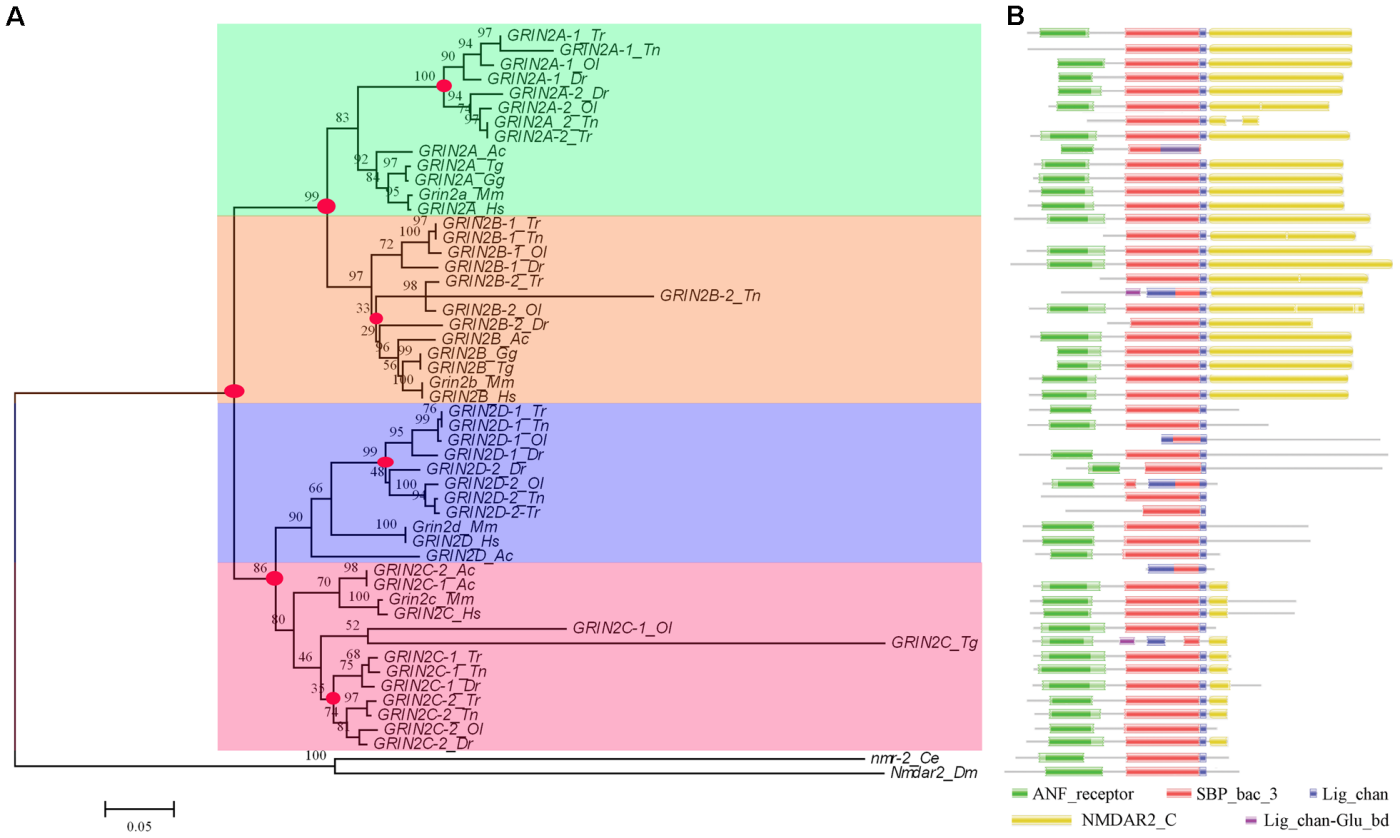
The phylogenetic tree (A) of NR2 proteins and their protein domain structure (B). The bootstrap consensus phylogenetic tree was constructed with the fruitfly Nmdar2 and nematode nmr-2 protein as outgroup using the neighbor-joining method in MEGA4, and the numbers indicate the percentage bootstrap support. The evolutionary distances were computed using the Poisson correction method and are in the units of the number of amino acid substitutions per site. Red circles indicate the gene duplication event supported by the phylogenetic analysis. Dm, *Drosophila melanogaster*; Ce, *Caenorhabditis elegans*; Dr, *Danio rerio*; Tr, *Takifugu rubripes*; Tn, *Tetraodon nigroviridis*; Ol, *Oryzias latipes*; Ac, *Anolis carolinensis*; Gg, *Gallus gallus*; Tg, *Taeniopygia guttata*; Mm, *Mus musculus*; Hs, *Homo sapiens*. Protein domains are shown as boxes based on identification by Pfam.

To investigate the evolutionary relationship among vertebrate NR2 subunit genes, whole protein sequences of fifty vertebrate NR2 subunit genes (exclude 2 pseudo genes and 1 not fully sequenced gene of 53 genes) and two invertebrate orthologues (include fruitfly *Nmdar2* and nematode *nmr-2*) were conducted in phylogenetic analysis. The analysis unambiguously defined the two vertebrate GRIN2 groups (designated as GRIN2A/B and GRIN2C/D group) with high bootstrap (Figure 1A). In the GRIN2A/B group, vertebrate GRIN2As and GRIN2Bs were classified into different clusters, the same as GRIN2Cs and GRIN2Ds in the GRIN2C/D group. These suggest that two rounds of duplication have occurred during the long term evolution of vertebrate GRIN2 genes. Interestingly, two anole lizard genes, GRIN2C-1 and GRIN2C-2, were clustered with vertebrate GRIN2C cluster. Each fishes have two GRIN2A genes, two GRIN2B genes, two GRIN2C genes and two GRIN2D genes. These suggest that an extra duplication event of NR2 subunit genes has occurred within the teleost lineage. Phylogenetic analysis showed that eight fish GRIN2A genes, eight fish GRIN2D genes form a monophyletic group with their respective tetrapod orthologue. However, eight GRIN2B genes and eight fish GRIN2C genes formed a sister clade with their respective tetrapod orthologue. From the phylogenetic tree, we can see that the Ensembl named tetraodon GRIN2A-1 was clustered with fish GRIN2A-2 cluster and tetraodon GRIN2A-2 was clustered with fish GRIN2A-1 cluster. In terms of evolutionary history, it should be proposed that the names of these two genes should be exchanged. Thus, we denominated these two gene name and some of chicken or teleost fishes gene name from the evolutionary point of view. It should be noted that the newly identified *GRIN2D-1_Ol* and *GRIN2D-2_Tr* could be successfully distinguished based on their phylogenetic relationships.

### Exon structure and conserved domain analysis

**Figure 2:**
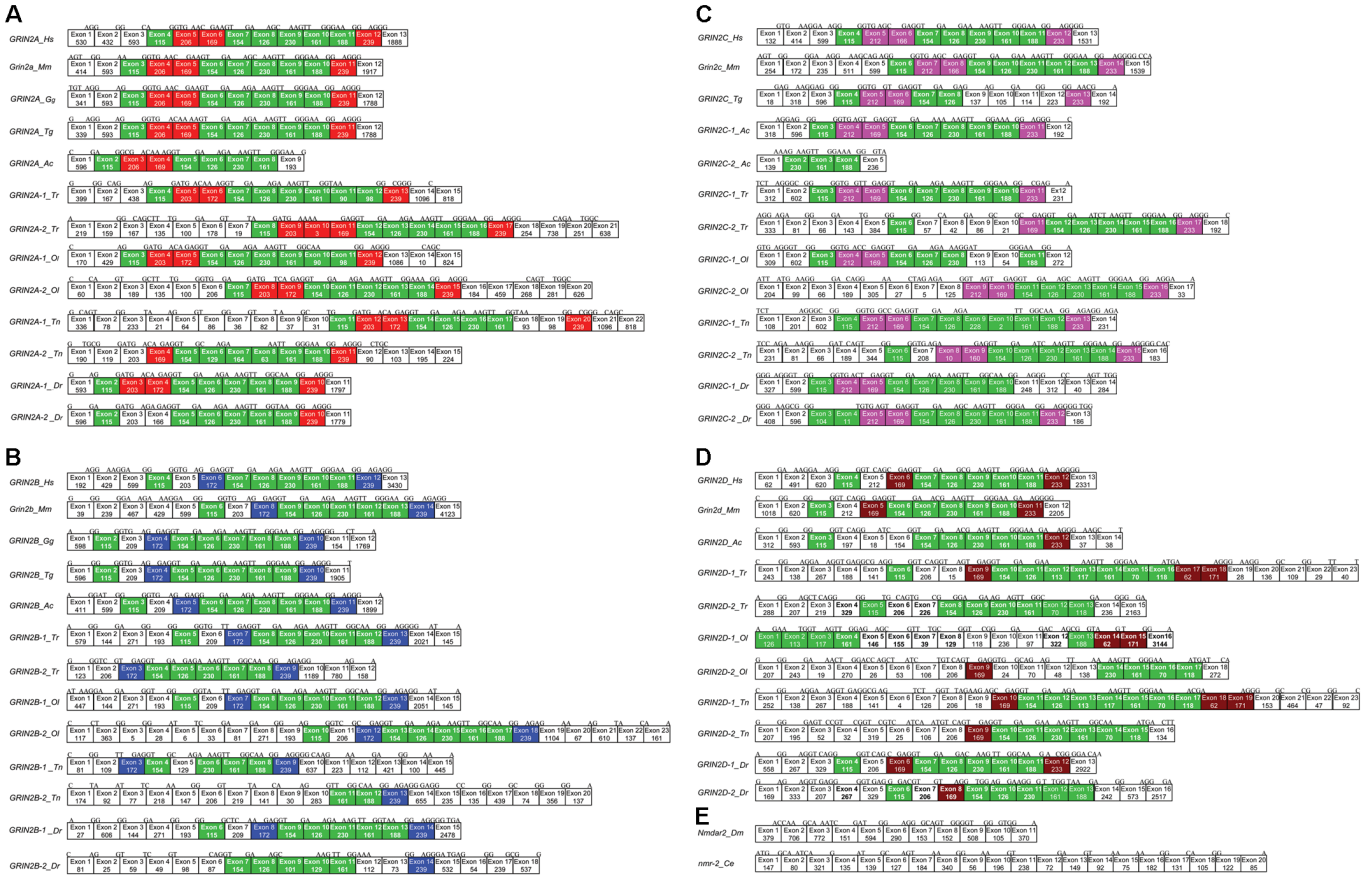
Exon structures (5′→3′) of vertebrate GRIN2A (A), GRIN2B (B), GRIN2C (C) and GRIN2D (D) genes and two invertebrate NR2 orthologues (E). The exons that transverse or flank the splice sites are indicated above each exon boundary. Numbers in boxes are nucleotide numbers. Exons coloured green are conserved in length among all NR2 subunit genes from all species. Exons coloured red, blue, pink or brown are conserved in length between each of GRIN2A, GRIN2B, GRIN2C, GRIN2D orthologues from all species. The size of each exon is not drawn to scale.

Conserved exon structures, including exons with the same numbers of nucleotides and the conserved intron phases, indicate similarities of the studied genes [26]. Two invertebrate NR2 subunit genes share no equal-length-exons with their vertebrate orthologues. However, similar splice phase were found in some invertebrate exons, such as 5? end of *Nmdar2_Dm* Exon 4, 5, 6 and 8. Forty-three of 50 vertebrate NR2 subunit genes share 4 exons with the same numbers of nucleotides and almost the same splice phase (Figure 2), including 154-nt exon, 126-nt exon, 230-nt exon, and 161-nt exon. These exons occurred in a region encoding highly conserved amino acid sequence among these nine species, which is the component of partial SBP_bac_3 domain (bacterial extracellular solute binding proteins family 3). In addition, some exons splitting were found. For example, Exon 11 and 12 of *GRIN2A-1_Tr* were split from 188-nt exon, Exon 9 and 10 of *GRIN2B-2_Dr* were split from 230-nt exon. In addition to the commonly shared exons with the same numbers of nucleotides mentioned above, there are equal-length-exons (Figure 2) shared by all orthologues of GRIN2A, GRIN2B, GRIN2C or GRIN2D genes, respectively. Interestingly, the exons structures of GRIN2A/GRIN2B were more closely related to each other than either to that of GRIN2C and GRIN2D, vice versa. The newly identified *GRIN2D-1_Ol* and *GRIN2D-2_Tr* share some similar exon structures with their orthologues.

For all NR2 subunit protein family members, SBP_bac_3 domain and Lig_chan (Ligand-gated ion channel) are present in all species with a high degree of similarity (Figure 1B), indicating the functional conservation features of NR2 subunit protein. The ANF_receptor domain (receptor family ligand binding region) nearly exists in all investigated species except for tetraodon GRIN2As and GRIN2Bs, fugu and zebra fish GRIN2B-2 and medaka GRIN2D. NMDAR2_C (N-methyl-D-aspartate receptor 2B3 C-terminus) domain disappeared in GRIN2D of all species and invertebrate NR2 orthologues, and protein sequence of NMDAR2_C domain partially retained in GRIN2C of all species except medaka. Our investigation indicated a functional divergence of NR2 subunit protein family members during the long evolution process.

### Paralogon analysis

**Figure 3:**
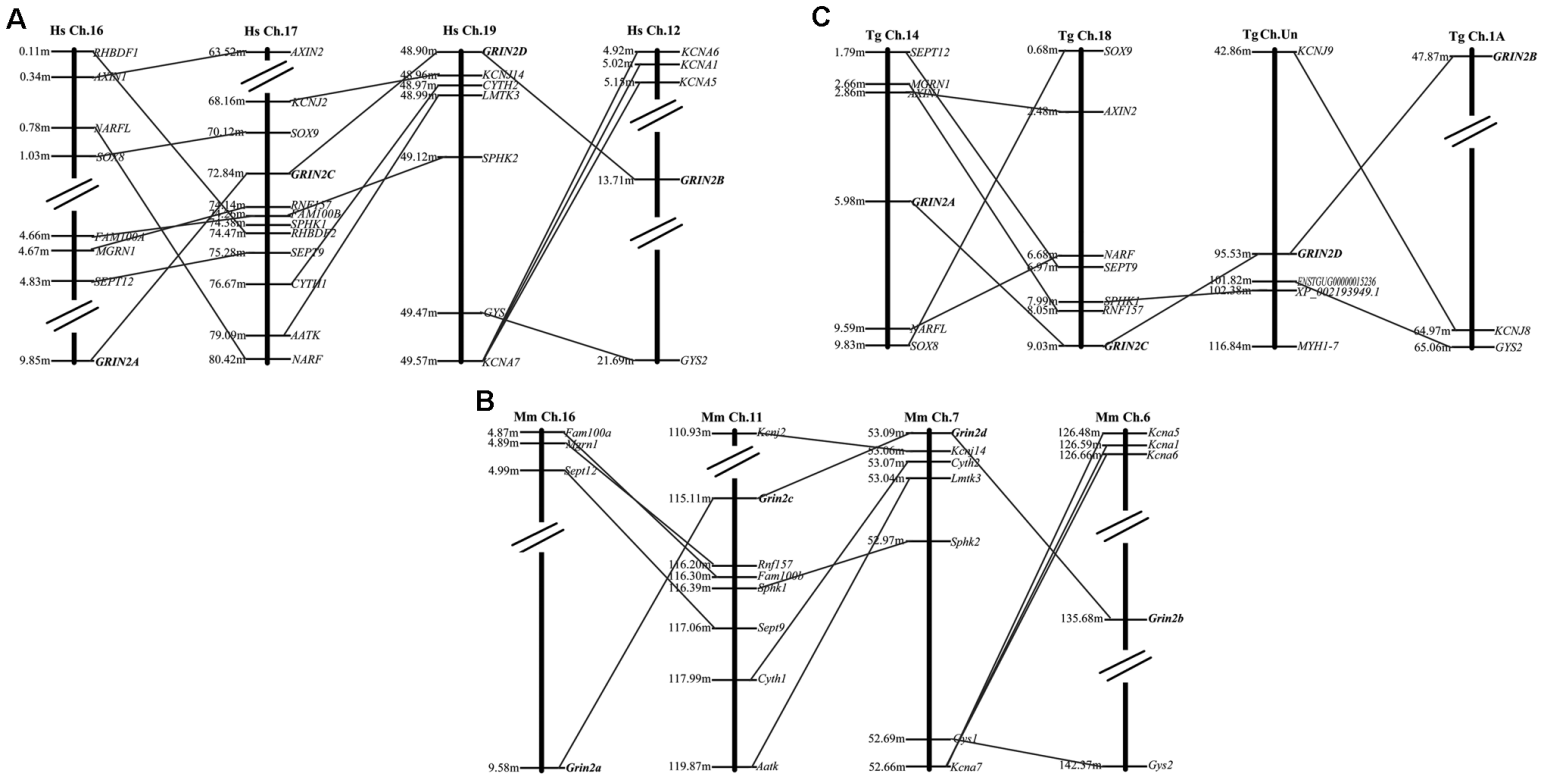
Paralogons surrounding the human (A), mouse (B), zebra finch (C) GRIN2A, GRIN2B, GRIN2C and GRIN2D genes. Within each connected block on each chromosome, genes are shown in actual order, although not to scale. Gene families are indicated by lines connecting the chromosomes. Paralogous genes flanking each GRIN2 genes share high degree of conservation among human, mouse and zebra finch.

The identification of chromosomal homologous segments within and between genomes (known as paralogons and syntenies, respectively) facilitates studying genome evolution, such as genome duplication and rearrangement [27]. Paralogous genes flanking each GRIN2 gene share high degree of conservation among human, mouse and zebra finch. Although we can hardly find the strictly linked gene with GRIN2 among all the four chromosomes, some linked gene with GRIN2 between chromosomes can be detected (Figure 3), suggesting the ancestral relationship of these chromosomes or chromosome fragments. As for human, seven paralogous neighbor was shared between GRIN2A and GRIN2C, four between GRIN2C and GRIN2D, and four between GRIN2D and GRIN2B. The order between these neighboring genes varied considerably, reflecting microinversions and other rearrangements.

When TBLASTN was used to interrogate the zebra finch genome, in ChrUn, we found a pseudo GRIN2D gene, 1 paralogous neighbor was shared between GRIN2C and GRIN2D, and 2 shared between GRIN2D and GRIN2B. Furthermore, a chicken GRIN2C gene was found using the similar method in Chromosome 18, and the protein sequences surrounding this gene shared high similarity with those surrounding chicken GRIN2A gene (data not shown).

### Syntenic analysis

**Figure 4:**
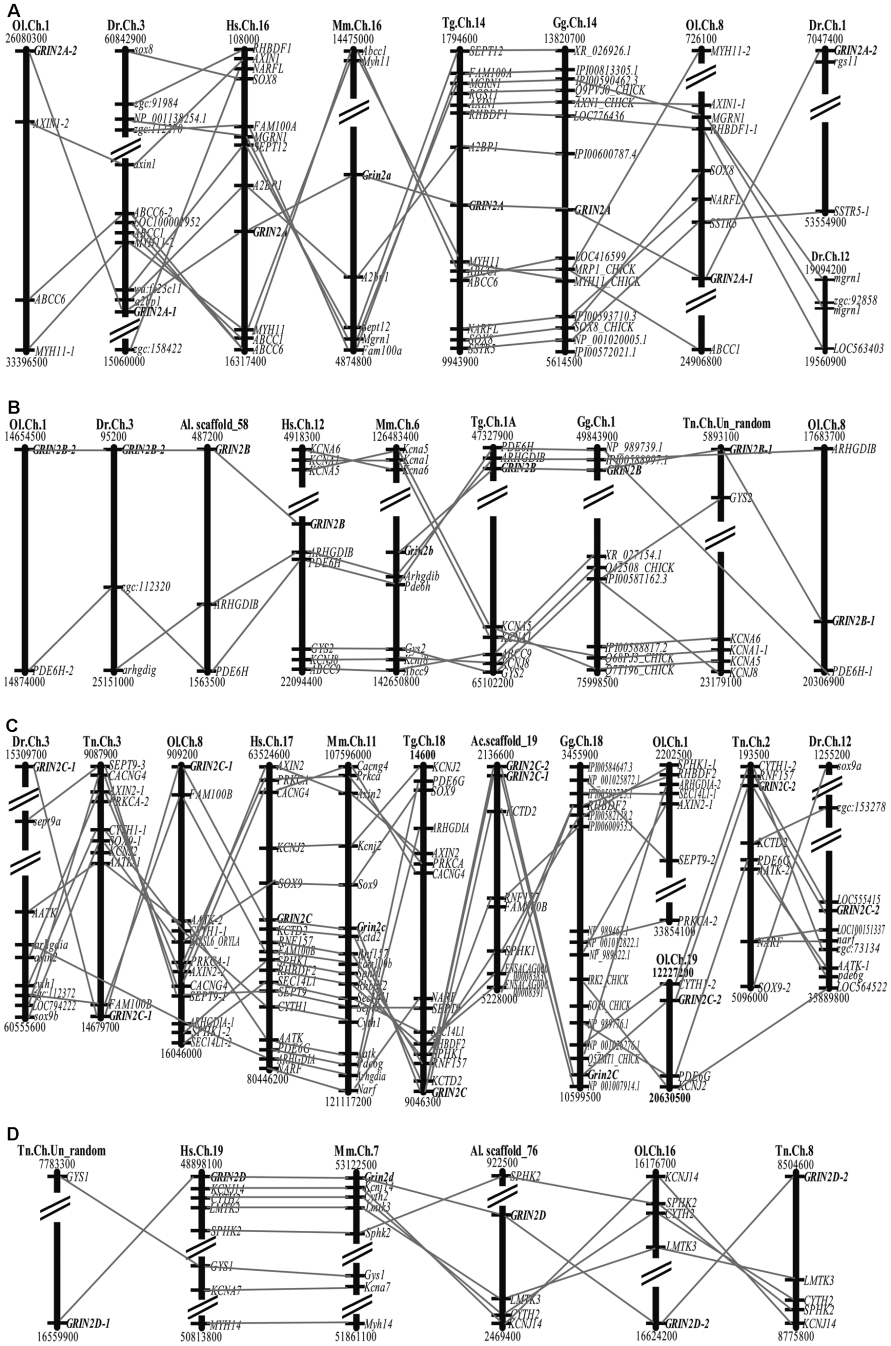
Comparison of genes surrounding GRIN2A (A), GRIN2B (B), GRIN2C (C) and GRIN2D (D) in chromosomes of different species. Orthologous genes flanking each GRIN2 genes define a synteny with high degree of conservation among human, mouse, zebra finch and chicken. (A) The tetrapod GRIN2A paralogon has two fish paralogons, the GRIN2A-1 paralogon and the GRIN2A-2 paralogon. AXIN1 on human chromosome 16, zebra finch chromosome 14 and chicken chromosome 14 near GRIN2A have two co-orthologs located in medaka chromosomes 1 and 8. The tetrapod GRIN2B paralogon has two fish paralogons, the GRIN2B-1 paralogon and the GRIN2B-2 paralogon (B). PDE6H on human chromosome 12, mouse chromosome 6, zebra finch chromosome 1A and chicken chromosome 1 near GRIN2B have two co-orthologs located in medaka chromosomes 1 and 8. (C) The tetrapod GRIN2C paralogon has two fish paralogons, the GRIN2C-1 paralogon and the GRIN2C-2 paralogon. Each of the SOX9, AATK and CYTH1 on human chromosome 17 near GRIN2C has two co-orthologs in zebrafish chromosomes 3 and 12 and, also, two co-orthologs in tetraodon chromosomes 2 and 3. Each GRIN2D paralogons (D) define a synteny with high degree of conservation among the studied tetrapod and fish species. The positions of genes on chromosomes are not drawn to scale.

Orthologous genes flanking each GRIN2 genes (Table S2) define a synteny with high degree of conservation among human, mouse, zebra finch and chicken. The tetrapod GRIN2A paralogon has two fish paralogons, the GRIN2A-1 paralogon and the GRIN2A-2 paralogon (Figure 4A). AXIN1 on human chromosome 16, zebra finch chromosome 14 and chicken chromosome 14 near GRIN2A have two co-orthologs located in medaka chromosome 1 and 8. Two fish paralogons, the GRIN2B-1 paralogon and the GRIN2B-2 paralogon, are accompanied by one tetrapod GRIN2B paralogon (Figure 4B). PDE6H, which is near GRIN2B on human chromosome 12, mouse chromosome 6, zebra finch chromosome 1A and chicken chromosome 1, have two co-orthologs located in medaka chromosome 1 and 8. The tetrapod GRIN2C paralogon has two fish paralogons, the GRIN2C-1 paralogon and the GRIN2C-2 paralogon (Figure 4C). Each of the SOX9, AATK and CYTH1 on human chromosome 17 near GRIN2C has two co-orthologs in zebrafish chromosomes 3 and 12 and, also, two co-orthologs in tetraodon chromosomes 2 and 3. Each GRIN2D paralogons define a synteny with high degree of conservation among the studied tetrapod and fish species (Figure 4D).

### Functional divergence analysis

Remarkably, we found (site-specific) altered selective constraint after the first gene duplications (GRIN2AB/GRIN2CD) was statistically significant. However, no statistically significant differences were found for two groups after the second duplication (GRIN2A/GRIN2B; GRIN2C/GRIN2D) (Table 1). To explore the pattern of type I functional divergence in each cluster, we performed functional distance (dF) analysis (Table 1). The functional branch length (bF) of GRIN2A, GRIN2B, GRIN2C and GRIN2D cluster was 0.4426, 0.3525, 0.7024 and -0.1207 (Table 1), respectively. Virtually zero bF of GRIN2D suggested GRIN2D cluster may contain a larger component of ancestral function compared to other gene clusters, and long bF of GRIN2A, GRIN2B and GRIN2C indicated substantial altered functional constraints in their gene cluster. These suggested site-specific altered selective constraints might contribute to most of the GRIN2 members leading to a group-specific functional evolution after their diversification.

**Table 1:** Type I functional divergence and purifying selection analysis for all GRIN2s.

Groups	θ±SE	LRT	*P*	Functional distance (dF)	Qk>0.67	Qk>0.9
2AB/2CD	0.629±0.082	59.182	<0.01	0.991	82	8
2A/2B	0.223±0.115	3.726	>0.05	0.252	1	0
2A/2C	0.789±0.120	43.184	<0.01	1.555	213	9
2A/2D	0.366±0.083	19.412	<0.01	0.455	7	2
2B/2C	0.695±0.104	44.648	<0.01	1.188	109	6
2B/2D	0.474±0.125	14.256	<0.01	0.642	14	0
2C/2D	0.038±0.133	0.081	>0.05	0.039	0	0
	**Negative site %** a		**Highly negative sites %** b		**Functional branch length (bF)**	
2A	40.21		33.52		0.4426	
2B	39.33		31.05		0.3525	
2C	30.22		22.71		0.7024	
2D	19.39		15.44		−0.1207	

Functional divergence (θ) for all pairwise comparisons of the GRIN2s were shown as value±standard error, the functional distance between two groups were calculated as –Ln(1-θ), and the functional branch length (bF) of each group were estimated by the least-squares method. Two hundred and twenty-four sites were investigated based on posterior probability (Qk) within GRIN2s.

a Percentages of sites under negative selection (*P*<0.1).

b Percentages of sites under strong negative selection (*P*<0.05).

Furthermore, the critical amino acid residues, responsible for the functional divergence, were predicted by calculating the site-specific profile based on posterior analysis for all pairs of groups with functional divergence (Table S3). For any two groups except GRIN2C/GRIN2D, there was at least one site that had the posterior probability higher than 0.67, and 4 pairs of groups had at least two sites with posterior probability higher than 0.90. For example, 82 amino acid residues with Qk>0.67 (eight of them with Qk>0.9) of total 224 sites were predicted to be responsible for type I functional divergence (site-specific rate difference) between GRIN2AB/GRIN2CD subfamilies, and all these 82 sites located at the Lig_chan (Ligand-gated ion channel) domain, including 18 at extracellular N-terminal transmembrane regions M1, 3 at M3 and 3 at re-entrant loop (M2).

### Natural selection analysis

None of the three positive selection models of PAML, M2, M3, and M8 predicted any site under positive selection for any GRIN2 gene with probabilities above 95% (data not shown). Also, the SLAC method predicted no positively selected site in these GRIN2 genes with probabilities above 95%. In search for negatively selected sites using SLAC method, we found that GRIN2A and GRIN2B have approximately similar percentages of negatively selected sites, almost twice the percentages of negatively selected sites of GRIN2D (Table 1) (Table S3).

## Discussion

Ohno's 2R hypothesis predicts the prevalence of quartet gene families descended by double duplication [17]. To date, the intensive controversies still surround this 2R hypothesis [28], [29], [30]. Our present study provides an intriguing subject for testing the hypothesis. Although suggested by Ryan T.J. that the four tetrapod NR2 subunits genes might arose from gene duplication events and adaptive evolution of NR2 subunits C-terminal cytoplasmic interaction domains has been chewed over [15], no further evidence has been provided and the exact evolutionary mode of them remains unclear. Our dendrogram analysis supports the hypothesis that the tetrapod GRIN2 gene family arose through genome duplication. Such conclusion presented here is further supported by the high degree of conservation of exon structures (Figure 2) and paralogon analysis (Figure 3). Each GRIN2 gene from both fish and tetrapods formed a separate clade suggests that two duplication events would have occurred after the divergence of the chordate ancestor from the ancestors of arthropods (only one GRIN2 gene), but before the divergence between bony fish and terrestrial vertebrates. It coincided with the time of two rounds of genome duplications that are postulated to have taken place [31], [32].

Although only three GRIN2 genes were found in the present chicken/zebra finch genome through extensive similarity-based search, two pseudo genes (*GRIN2D_Tg* and *GRIN2D_Gg*) were identified. Such observation highlights the “one-to-four” evolution mode descended by double duplication. Five GRIN2 genes, including two GRIN2C paralogs, GRIN2C-1 and GRIN2C-2, were found in present anole lizard genome. GRIN2C-1 and GRIN2C-2 reside in the same chromosome with close location (0.017 M) and the DNA sequence of GRIN2C-2 share high similarity (>99%) with that of GRIN2C-1. Two paralogs located in tandem in a chromosomal segment are likely the result of single-gene duplication [33]. Therefore, it should be tandem GRIN2C replication occurred in the lizard genome.

Each of the four teleost fishes has two GRIN2A genes, two GRIN2B genes, two GRIN2C genes and two GRIN2D genes (Figure 1A). In support of the phylogenetic analysis results, the exon structure analysis showed that 27 of the 32 teleost fish GRIN2 genes share four equal-length-exons with conserved intron phases, indicating the evolutionary conservation of these teleost GRIN2 genes. Conserved syntenic regions would provide remarkable insight into the evolutionary paths of genes and genomes [21], [22], [34], [35]. One to two (tetrapod to fish) conserved syntenies defined by genes flanking GRIN2A or GRIN2C and their orthologs or co-orthologs clearly exist in medaka, tetraodon and zebrafish (Figure 4). It is suggested the most parsimonious evolutionary trajectory for occurrences of these extra copies of teleost GRIN2 genes is from an ancient whole-genome duplication event, which occurred in the teleost lineage after its divergence from the tetrapod lineage but before its radiation [20], [22], [23], [28]. In addition although we can hardly find “one-to-two” co-orthologs near GRIN2D, each GRIN2D paralogon defines a synteny with high degree of conservation among the studied tetrapod and fish species. Fish-specific whole genome duplication has been used to explain the evolution of fish genes and gene families [36], [37], [38]. Thus, in-depth analysis of teleost GRIN2s evolution provides new evidence for 3R hypothesis.

**Figure 5:**
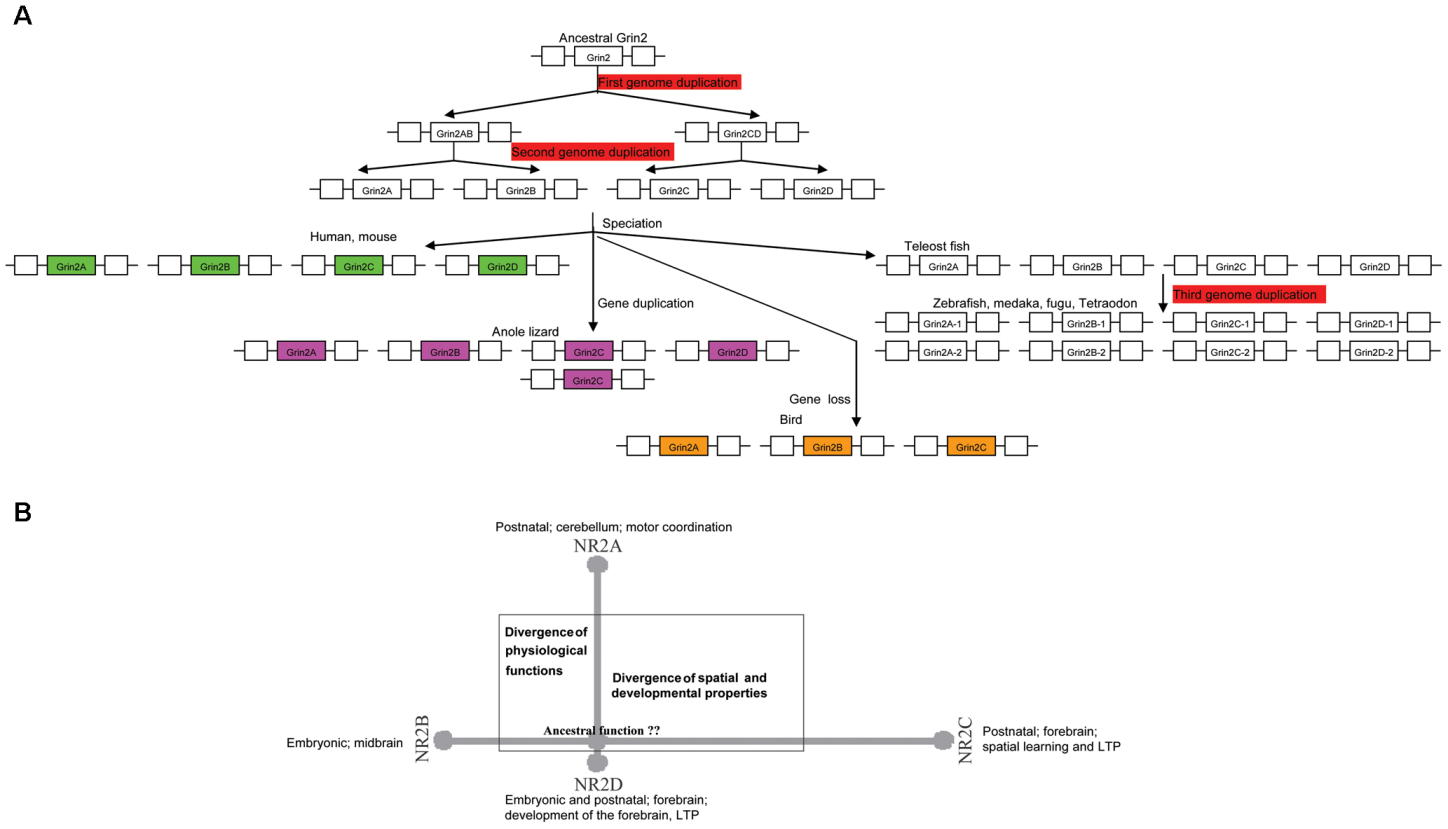
Evolutionary mode and functional divergence of the vertebrate GRIN2s genes.

Another interesting finding of this study is that our dendrogram analysis indicates two vertebrate GRIN2 groups (Figure 1A). It is likely that, during the first-round genome duplication of chordate evolution, the ancestral chordate GRIN2 gene gave rise to GRIN2AB and GRIN2CD. Besides, during the successive second round genome duplication, GRIN2AB gave rise to GRIN2A and GRIN2B, while GRIN2CD gave rise to GRIN2C and GRIN2D (Figure 5A). Finally, the tetrapod lineage has maintained GRIN2A, GRIN2B, GRIN2C and GRIN2D. However, tandem replication was occurred in lizard, leading to a total of five GRIN2 genes in present anole lizard. During the third-round genome duplication occurred in the teleost lineage, GRIN2A gave rise to GRIN2A-1 and GRIN2A-2, GRIN2B gave rise to GRIN2B-1 and GRIN2B-2, GRIN2C gave rise to GRIN2C-1 and GRIN2C-2, and GRIN2D gave rise to GRIN2D-1 and GRIN2D-2, which have been preserved in all of the studied teleost fishes.

In addition, on the basis of phylogenetic analysis, we found that formerly named tetraodon GRIN2A-1 and GRIN2A-2 were clustered with fish GRIN2A-2 and GRIN2A-1 cluster, respectively. Accordingly, names of these two genes were exchanged from the evolutionary point of view. Also, zebra fish A2BIF1_DANRE, grin2ab, NP_001121809.1, LOC100147922, sidkey-154b17.1, LOC100149482 were be renamed as GRIN2A-1, GRIN2A-2, GRIN2B-1, GRIN2C-2, GRIN2D-1, GRIN2D-2 respectively. Chicken IPI00599444.2, IPI00599161.2 were renamed as GRIN2A, GRIN2B, respectively. The new names may be well reflect the historic context and facilitate the correct genome annotation of these genes. Synteny between or among the chromosomes of a species or several species can aid in identifying homologous genes [39]. Based on similarity-based research, we documented a putative GRIN2C gene residing in the chicken Chromosome 18. The protein sequences surrounding this gene shared high similarity with those surrounding chicken GRIN2A gene. Genes flanking this putative GRIN2C gene show a high degree of conservation among those flanking human, mouse and zebra finch GRIN2C gene. However, due to the sequenced gap, only 7 exons were obtained in this novel *GRIN2C_Gg* gene.

After gene duplication, two duplicates can undergo substantial functional divergence, and it plays a major role in the evolution of new functions [40]. Discrete physiological functions and developmental properties were observed in the present NR2 genes [41], [42], [43], [44] (Figure 5B). Namely, NR2B, expressed both embryonically and postnatally throughout the forebrain, play an important role in the development [41]; NR2A is postnatally expressed throughout the forebrain and knockout of NR2A leading to a deficiency in spatial learning and LTP [42]; NR2C is postnatally restricted to the cerebellum, knockout of NR2C results in a decrease in NMDA receptor mediated currents in the granular cells of the cerebellum, and NR2A/NR2C double knockouts result in impaired motor coordination [43], [44]; NR2D is found embryonically in the midbrain. It has previously been observed that an unstructured but modular intracellular domain of NR2 C-terminus parallels the expansion in complexity of an NMDA receptor signaling complex in the vertebrate lineage. We also observed disappeared or truncated C-terminus NMDAR2_C domain in GRIN2D or GRIN2C, respectively. In our study, site-specific altered selective constraint after the first gene duplication (GRIN2AB/GRIN2CD) is statistically significant, while no statistical significant differences for two groups after the second duplication (GRIN2A/GRIN2B; GRIN2C/GRIN2D) (Table 1). Furthermore, 82 amino acid residues with Qk>0.67 (eight of them with Qk>0.9) were predicted to be responsible for type I functional divergence between GRIN2AB/GRIN2CD subfamilies, and all these 82 sites located at the N-terminal Lig_chan domain, including 18 at extracellular N-terminal transmembrane regions M1, 3 at M3 and 3 at re-entrant loop (M2). Virtually zero bF of GRIN2D indicates that the evolutionary rate of each site in this duplicate gene is almost identical to the ancestral gene. Long bF of GRIN2A, GRIN2B and GRIN2C indicated substantial altered functional constraints in their gene cluster. Our results suggested 1)the functions of the present NR2 genes were subsequently acquired; 2)site-specific altered selective constraints might contribute to most of the GRIN2 members leading to a group-specific functional evolution after their diversification, amino acid residues located at the N-terminal Lig_chan domain were responsible for type I functional divergence between these GRIN2 subfamilies; 3) purifying selection has been the prominent natural pressure operating on these diversified GRIN2 genes. In previous research, the role of duplication of the elongated vertebrate NR2 in brain morphology and cognitive function has been discussed at the level of anatomy and signal transduction complexes [15], [45]. This indicates that extra GRIN2 genes might be of benefit to the teleost species for their adaptation to diverse ecological niches through modified brain and cognitive function including learning and memory [15], [45].

In summary, two-round genome duplication generated quartet GRIN2 genes and the third-round fish-specific genome duplication generated extra copies of fish GRIN2 genes. Functional divergence of NR2 genes mostly occurred at the first-round duplication. Amino acid residues located at the N-terminal Lig_chan domain were responsible for type I functional divergence between these GRIN2 subfamilies and purifying selection has been the prominent natural pressure operating on these diversified GRIN2 genes. Collectively, these findings provide intriguing subjects for testing the 2R and 3R hypothesis, and we expect it could provide more insights into the underlying evolution mechanisms of cognition in vertebrate.

## Materials and Methods

### Data sets and phylogenetic analysis

Human GRIN2A, GRIN2B GRIN2C and GRIN2D were used as the query sequences in BLASTP and TBLASTN searches (E<1e-10) against NCBI and Ensembl databases (March 2010) of human, mouse, zebra finch, chicken, anole lizard, zebrafish, medaka, tetraodon and fugu. Each matching sequence was annotated by Ensembl (http://www.ensembl.org), GenBank (http://www.ncbi.nlm.nih.gov) or Genscan (http://genes.mit.edu/GENSCAN.html). Exon boundaries within the coding regions of each NR2 subunit genes were determined according to Ensembl, GenBank or GenScan. Multiple sequence alignments of NR2 subunits were generated using ClustalW [46] with the default setting. The bootstrap consensus phylogenetic tree inferred from 1000 replicates was constructed using the neighbor-joining method with MEGA4 [47].

### Paralogon analysis within human, mouse and zebra finch

All the predicted genes within 20 Mb of each human, mouse and zebra finch NR2 subunit paralogs were obtained using the BioMart mode in Ensembl (March 2010). Only genes belonging to the same putative gene family (with protein sequences showed similarities above 50% and supported by Ensembl paralogs prediction) within each species were regarded as paralogs. Genes exhibiting orthologous relationship in two or three species (human/mouse/zebra finch) and supporting by phylogenetic analysis were selected for paralogon analysis.

### Syntenic analysis

Neighboring genes flanking the chicken, anole lizard, zebrafish, medaka, fugu and tetraodon NR2 paralogs were also obtained using the BioMart mode in Ensembl from dataset of the chicken (Ensembl WASHUC2), anole lizard (Ensembl AnoCar1.0), zebrafish (Ensembl Zv8), medaka (Ensembl HdrR) fugu (Ensembl FUGU 4.0) and tetraodon (Ensembl TETRAODON8.0) genome, respectively. Then, they were compared with the selected genes through paralogon analysis as described above. From the BioMart output tables, we only selected the genes that exhibit one to two (tetrapod to zebrafish or medaka or fugu or tetraodon) orthologies supporting by phylogenetic analysis.

### Analysis of Type I functional divergence

Previous study indicated that type I functional divergence after gene duplication leads to altered selective constraints between duplicate genes despite the underlying evolutionary mechanisms [48]. The DIVERGE [49] program was employed to examine whether one of the duplicates has evolved at an accelerated rate following the duplication. The potential domains of NR2 subunit protein was identified based on the Pfam database [50] to better understand the conserved exons and depict their role in the functional divergence of NR2 subunits proteins.

### Analysis of positive selection

The coding sequences of the tetrapod and fish NR2 subunits genes were aligned according to the amino acid alignment by CodonAlign 2.0 (http://www.sinauer.com/hall/2e/). Several site-specific models (M0, M1, M2, M3, M7 and M8) were use to detect positive selection using the codeml program implemented in PAML [51]. We also applied the tests of positive and negative selection from coding sequence alignments [52] implemented as the Single Likelihood Ancestor Counting (SLAC) analysis in the datamonkey web server [53].

## Supporting Information

Table S1 NR2 subunit genes in different species. Chicken GRIN2C gene was identified to reside in 10.46 m of chicken Chromosome 18, because of the sequenced gap of this location, only 7 exons was obtained using GENSCAN prediction.
(0.03 MB XLS)

Table S2 Orthologous genes surrounding each Grin2 gene in chromosomes of different species.
(0.08 MB XLS)

Table S3 Site of Type I functional divergence and purifying selection. Two hundred and twenty-four sites were investigated based on posterior probability (Qk) within GRIN2s. Sites with Qk>0.67 or Qk>0.9 were listed relative to human GRIN2A protein sequence. Sites located at N-terminal transmembrane regions M1, M3 or re-entrant loop (M2) were colored red, green or pink, respectively. Sites under negative selection (P<0.1) or strong negative selection (P<0.05) were listed relative to human GRIN2s protein sequences, respectively.
(0.19 MB DOC)
