# Supplementary material for: Evolutionary Mode and Functional Divergence of Vertebrate NMDA Receptor Subunit 2 Genes
Source: PLoS One. 2010 Oct 14;5(10):e13342. doi: 10.1371/journal.pone.0013342 (PMC2954789; doi:10.1371/journal.pone.0013342)
Supplement: Table S3 — Site of Type I functional divergence and purifying selection. Two hundred and twenty-four sites were investigated based on posterior probability (Qk) within GRIN2s. Sites with Qk>0.67 or Qk>0.9 were listed relative to human GRIN2A protein sequence. Sites located at N-terminal transmembrane regions M1, M3 or re-entrant loop (M2) were colored red, green or pink, respectively. Sites under negative selection (P<0.1) or strong negative selection (P<0.05) were listed relative to human GRIN2s protein sequences, respectively. (0.19 MB DOC) [file pone.0013342.s003.doc]

**Table S3 Site of Type I functional divergence and purifying selection**

| **Groups** | **Qk>0.67** | | **Qk>0.9** |
| --- | --- | --- | --- |
| 2AB/2CD | 552P,555A,556S,**557V,558W,559V,560M,561M,562F,563V,564M,565L,567I,568V,569S,570A,572A,573V,574F,575V,576F**,577E,580S,581P,582V,584Y,585N,588L,589A,598S,**601I,610G,611L**,619V,**629I,630M,633V**,657E,658F,659V,669K,677S,679P,680F,681R,684T,685V,686P,687N,689S,691E,692R,693N,694I,695R,697N,698Y,701M,702H,704Y,705M,714E,717L,719S,721K,738K,743E,744G,745C,746K,752S,753G,754V,755I,758T,765L,766Q,774Q,779L,781Q,784G,787E | | **568V**,589A,598S,633V,692R,714E,719S,781Q |
| 2A/2B | 718V | | no |
| 2A/2C | 552P,555A,556S,557V,558W,559V,560M,561M,562F,563V,564M,565L,566L,567I,568V,569S,570A,571I,572A,573V,574F,575V,576F,577E,578Y,579F,580S,581P,582V,583G,584Y,585N,586R,587N,588L,589A,591G,596G,597P,598S,599F,600T,601I,602G,603K,604A,605I,606W,607L,608L,609W,610G,611L,612V,613F,614N,615N,616S,617V,618P,619V,620Q,621N,622P,623K,624G,626T,627S,628K,629I,630M,631V,632S,633V,634W,635A,636F,637F,638A,639V,640I,641F,642L,643A,644S,645Y,646T,647A,649L,650A,651A,652F,653M,654I,655Q,656E,657E,658F,659V,660D,661Q,662V,663T,664G,665L,666S,667D,668K,669K,670F,671Q,676Y,677S,678P,679P,680F,681R,682F,683G,684T,685V,686P,687N,688G,689S,690T,691E,692R,693N,694I,695R,696N,697N,698Y,699P,701M,702H,704Y,705M,707K,709N,710Q,712G,713V,714E,715D,716A,717L,718V,719S,720L,721K,723G,724K,725L,726D,727A,728F,729I,730Y,731D,732A,733A,734V,735L,736N,737Y,738K,739A,740G,741R,742D,743E,744G,745C,746K,747L,748V,749T,750I,751G,752S,753G,754V,755I,756F,757A,758T,762G,763I,764A,765L,766Q,767K,768G,769S,771W,772K,773R,774Q,775I,776D,777L,778A,779L,780L,781Q,782F,783V,784G,785D,786G,787E | | 575V,598S,605I,669K,714E,715D,718V,743E,781Q |
| 2A/2D | 575V,712G,714E,715D,718V,774Q,781Q | | 714E,718V |
| 2B/2C | 552P,555A,556S,557V,558W,559V,560M,561M,562F,563V,564M,565L,567I,568V,569S,570A,572A,573V,574F,575V,576F,577E,578Y,579F,580S,581P,582V,584Y,585N,588L,589A,598S,605I,610G,611L,619V,633V,639V,640I,641F,642L,643A,644S,645Y,646T,647A,649L,650A,651A,652F,653M,654I,656E,657E,659V,660D,661Q,662V,665L,666S,667D,668K,669K,671Q,674H,676Y,677S,678P,679P,680F,681R,682F,683G,684T,685V,686P,687N,688G,689S,690T,691E,692R,693N,694I,695R,696N,697N,698Y,701M,702H,704Y,705M,708F,713V,714E,716A,717L,719S,720L,721K,723G,754V,755I,758T,763I,766Q,781Q,784G,787E | | 559V,567I,568V,598S,669K,674H |
| 2B/2D | 559V,568V,571I,575V,589A,598S,633V,668K,674H,681R,714E,719S,766Q,774Q | | no |
| 2C/2D | no | | no |
|  | **Negative sites** | **Highly negative sites** | |
| 2A | 6Y,11V,32P,48E,52R,70V,79P,80K,87C,89L,96H,102D,105D,110A,125L,129G,130G,132S,134I,139D,142S,143T,147F,148G,149A,154Q,155A,159L,165Y,168H,170F,171S,172L,173V,174T,178P,179G,180Y,181R,183F,184I,190T,194S,207D,208T,212D,220K,224S,225S,226V,227I,229L,231C,233K,234D,235E,239I,242E,243A,245S,246L,247G,248L,250G,251Y,252D,253F,256I,258P,260L,264N,266E,269P,272F,273P,275G,277I,278S,281Y,282D,290A,291R,293R,294D,296I,298I,299L,304S,306M,308E,309K,313I,314P,315E,323Q,339V,340N,341V,342T,345G,346K,347D,348L,349S,351T,352E,353E,354G,355Y,356Q,361L,364I,365V,367N,370R,371E,373E,376G,377K,379E,380N,382T,387H,388A,391P,392R,393Y,396F,397S,398D,401P,402D,406L,408I,410T,411L,413E,414A,415P,416F,418I,419V,420E,421D,423D,425L,426T,427E,428T,429C,433T,435P,436C,437R,438K,439F,446T,448E,449G,451N,452V,453K,457K,458G,461I,462D,463I,464L,465K,466K,467L,468S,469R,471V,473F,474T,475Y,477L,478Y,479L,480V,481T,482N,483G,484K,485H,486G,487K,488K,490N,491N,494N,495G,497I,498G,501V,502Y,503Q,504R,505A,506V,508A,509V,510G,511S,512L,513T,514I,516E,518R,519S,522V,525S,527P,528F,529V,530E,531T,532G,533I,534S,535V,537V,538S,539R,540S,541N,542G,543T,544V,545S,546P,547S,548A,549F,550L,551E,552P,553F,554S,555A,556S,557V,559V,562F,563V,565L,566L,568V,570A,571I,572A,573V,574F,576F,577E,578Y,579F,580S,581P,582V,583G,584Y,586R,588L,589A,590K,591G,593A,594P,595H,596G,597P,598S,599F,600T,601I,602G,603K,604A,605I,607L,608L,611L,612V,613F,615N,616S,618P,619V,620Q,622P,623K,624G,625T,627S,628K,629I,630M,631V,632S,633V,635A,637F,638A,640I,643A,644S,646T,647A,648N,650A,652F,654I,655Q,657E,659V,660D,661Q,662V,663T,664G,665L,668K,670F,671Q,672R,673P,674H,676Y,677S,678P,679P,680F,681R,682F,683G,684T,685V,686P,687N,688G,690T,691E,692R,693N,694I,695R,696N,698Y,699P,702H,707K,708F,709N,710Q,711K,712G,713V,715D,716A,720L,722T,723G,724K,725L,726D,727A,729I,730Y,731D,732A,733A,734V,735L,736N,737Y,739A,740G,741R,743E,745C,746K,747L,748V,749T,750I,751G,752S,753G,756F,757A,758T,759T,760G,761Y,762G,763I,764A,765L,766Q,767K,768G,769S,772K,773R,775I,776D,777L,778A,779L,780L,782F,783V,784G,785D,787E,789E,791L,792E,793T,797T,798G,799I,800C,801H,802N,803E,805N,806E,810S,811Q,813D,818A,819G,820V,821F,824L,825A,829A,832L,833I,835F,837W,838E,839H,841F,844K,845L,848C,849F,850T,851G,853C,854S,855D,856R,857P,858G,859L,860L,863I,866G,867I,869S,870C,871I,872H,873G,874V,875H,876I,877E,879K,880K,885F,888T,890S,891Q,895L,896K,897L,898L,900S,901A,902K,907M,909N,914R,917S,920R,926Q,930L,937D,938K,939G,940N,943Y,952K,962E,970R,972K,975L,976N,979V,980F,982G,983Q,987T,993P,994N,999A,1002T,1008S,1009R,1010P,1011R,1013L,1016K,1017S,1020S,1021I,1022R,1023Q,1047K,1048S,1050R,1051Y,1052L,1053P,1054E,1059S,1060D,1062S,1064T,1065S,1067R,1076N,1081K,1084D,1088R,1095P,1101V,1107K,1113P,1116K,1123E,1131D,1141L,1142P,1143E,1153D,1157N,1164T,1165L,1171P,1172L,1183Q,1184Y,1187Y,1189K,1207Y,1208R,1210N,1212T,1213H,1214C,1215R,1217C,1218L,1219S,1220N,1224Y,1226G,1228F,1231R,1232S,1233P,1235K,1236C,1238A,1239C,1245L,1246Y,1248I,1251D,1254L,1264E,1267Y,1269Q,1273Q,1276A,1280Q,1281K,1283K,1290H,1294N,1295I,1298K,1302L,1303D,1306R,1307P,1311I,1314K,1317E,1318R,1325Y,1326G,1329F,1353R,1355K,1359P,1361H,1365N,1366P,1367F,1377L,1380G,1381R,1387Y,1388K,1389H,1390S,1402R,1415D,1428V,1437N,1443R,1450N,1452R,1454Y,1457M,1458P,1459S,1460I,1462S | 11V,32P,70V,79P,80K,87C,96H,110A,125L,129G,130G,132S,134I,139D,142S,143T,147F,148G,149A,155A,159L,165Y,168H,170F,171S,173V,174T,178P,179G,180Y,183F,184I,190T,207D,212D,220K,226V,227I,229L,231C,235E,239I,245S,247G,248L,250G,252D,256I,258P,260L,266E,269P,272F,273P,275G,277I,278S,282D,290A,291R,293R,294D,296I,298I,299L,304S,306M,309K,315E,323Q,339V,340N,341V,345G,346K,347D,349S,351T,354G,355Y,356Q,361L,364I,365V,367N,370R,371E,373E,376G,377K,379E,380N,382T,387H,388A,391P,392R,396F,398D,401P,402D,406L,408I,410T,411L,413E,415P,416F,418I,419V,420E,423D,425L,426T,427E,428T,429C,433T,435P,436C,437R,439F,449G,451N,453K,457K,458G,461I,462D,463I,464L,465K,466K,467L,468S,471V,473F,474T,475Y,477L,478Y,479L,480V,481T,482N,483G,484K,485H,486G,487K,488K,490N,491N,494N,495G,497I,498G,501V,503Q,504R,505A,506V,508A,509V,510G,511S,513T,518R,519S,522V,525S,527P,528F,529V,530E,531T,532G,534S,535V,537V,538S,539R,540S,541N,542G,543T,544V,545S,546P,547S,548A,549F,550L,551E,552P,553F,554S,555A,556S,557V,559V,562F,563V,565L,566L,568V,570A,571I,572A,573V,574F,576F,577E,578Y,579F,580S,581P,583G,584Y,588L,589A,590K,591G,593A,594P,595H,596G,597P,598S,599F,600T,602G,603K,604A,605I,607L,608L,611L,612V,613F,615N,616S,618P,619V,620Q,622P,623K,624G,625T,627S,628K,629I,630M,631V,632S,633V,635A,637F,638A,643A,644S,646T,647A,648N,650A,652F,655Q,657E,659V,660D,661Q,662V,663T,664G,668K,670F,671Q,672R,673P,677S,678P,679P,680F,681R,682F,683G,684T,685V,686P,687N,688G,690T,691E,692R,693N,695R,696N,698Y,702H,707K,709N,710Q,712G,713V,715D,716A,720L,722T,723G,724K,726D,727A,730Y,731D,732A,733A,734V,735L,736N,737Y,739A,740G,741R,743E,745C,746K,748V,749T,750I,751G,752S,753G,756F,757A,758T,759T,760G,761Y,762G,763I,764A,765L,766Q,767K,768G,769S,772K,773R,775I,776D,777L,778A,779L,780L,782F,783V,784G,785D,787E,789E,791L,792E,793T,797T,798G,799I,800C,801H,802N,803E,805N,806E,810S,811Q,813D,818A,819G,820V,821F,825A,829A,833I,835F,837W,838E,839H,841F,845L,848C,849F,850T,851G,853C,855D,857P,858G,859L,860L,866G,867I,869S,870C,871I,872H,873G,874V,875H,876I,879K,880K,885F,888T,890S,891Q,895L,896K,897L,898L,900S,901A,902K,907M,909N,914R,917S,920R,926Q,937D,938K,939G,952K,962E,970R,972K,975L,976N,979V,980F,983Q,993P,999A,1008S,1010P,1011R,1013L,1016K,1017S,1020S,1021I,1022R,1023Q,1047K,1050R,1051Y,1052L,1053P,1054E,1059S,1060D,1062S,1064T,1065S,1067R,1076N,1081K,1084D,1088R,1095P,1116K,1123E,1131D,1142P,1143E,1157N,1164T,1171P,1172L,1187Y,1189K,1207Y,1208R,1212T,1213H,1214C,1215R,1217C,1218L,1219S,1220N,1231R,1232S,1233P,1236C,1238A,1239C,1248I,1251D,1254L,1267Y,1273Q,1280Q,1283K,1294N,1302L,1303D,1306R,1307P,1311I,1314K,1318R,1326G,1353R,1355K,1359P,1361H,1365N,1366P,1367F,1381R,1387Y,1388K,1402R,1415D,1428V,1443R,1450N,1452R,1457M,1458P,1459S,1462S | |
| 2B | 14L,19L,34S,36G,37I,41L,47E,50I,60H,61H,63S,65V,69E,76T,78P,81I,95Q,99F,102D,103T,106E,108I,109A,113D,116S,117A,119T,123I,125G,127H,130S,144F,145Q,146F,147G,148P,150I,154A,156V,157M,159N,163E,164Y,169F,171I,173T,174T,175Y,177P,178G,186I,193S,198E,201E,202V,203L,205L,208S,212G,214S,217Q,218N,219Q,222K,223L,224Q,225S,228I,229L,230L,233T,236E,241F,246S,247V,248G,250T,255T,259P,264G,270P,273F,274P,276G,280V,281S,283D,284E,287Y,291A,292R,293V,294R,295D,296G,299I,300I,303A,304A,305S,310E,312S,318K,319S,321C,327K,335L,338Y,340I,341N,343T,344F,345E,346G,347R,348N,349L,350S,352S,353E,354D,355G,359H,360P,361K,362L,363V,364I,365I,367L,368N,371R,375R,376V,378K,384L,387K,388Y,389Y,392P,393R,395C,401Q,405H,408I,410T,411L,413E,414A,415P,418I,420E,423D,424P,426S,430M,431R,432N,433T,434V,435P,436C,437Q,438K,440I,444N,446T,449E,454K,456C,457C,458K,459G,460F,461C,462I,469S,472V,473K,474F,477D,478L,481V,484G,486H,487G,491N,492G,493T,496G,498I,501V,504K,507Y,508M,509A,511G,512S,513L,515I,517E,518E,519R,520S,521E,522V,523V,525F,526S,527V,528P,529F,530I,532T,534I,535S,536V,538V,539S,540R,541S,542N,544T,545V,550F,552E,553P,555S,557D,558V,561M,563F,564V,567L,571A,573A,574V,575F,577F,581S,584G,585Y,587R,588C,590A,591D,594E,597G,598P,602I,603G,604K,605A,608L,609L,612L,613V,614F,615N,617S,618V,619P,620V,622N,624K,625G,627T,628S,629K,632V,633S,638F,640V,641I,642F,649N,650L,652A,656Q,658E,659Y,665G,666L,668D,669K,671F,672Q,674P,675N,677F,678S,679P,680P,681F,682R,683F,684G,685T,686V,687P,688N,689G,690S,691T,692E,693R,694N,695I,696R,697N,699Y,703H,705Y,710N,711Q,713G,714V,716D,717A,718L,720S,722K,724G,725K,726L,727D,728A,729F,730I,731Y,732D,733A,734A,735V,737N,740A,742R,743D,746C,751I,753S,754G,757F,758A,760T,761G,762Y,765A,767Q,768K,769D,770S,773K,774R,775Q,777D,778L,779A,780I,781L,783L,784F,786D,787G,791E,794A,795L,797L,798T,801C,802H,803N,806N,808V,814D,819A,820G,821V,823Y,825L,826G,827A,830A,837I,839E,840H,842F,843Y,847R,850F,852G,854C,855S,856G,857K,858P,859G,866R,867G,868I,871C,872I,873H,874G,875V,877I,878E,879E,882S,883V,885N,886S,887P,888T,889A,894T,895H,897N,898I,900R,901L,902L,903R,904T,906K,909A,911L,912S,914V,915N,916G,917S,918P,919Q,920S,921A,922L,925I,926R,927R,928E,929S,930S,931V,933D,934I,936E,937H,938R,940S,941F,942T,943H,944S,947K,948S,951N,952P,955E,957N,958L,960S,961D,962Y,963I,964S,966V,967E,969T,970F,971G,972N,973L,978S,979N,980V,982Q,983D,984H,985Y,987H,989H,991P,992H,993S,994I,995G,998S,1001D,1002G,1004Y,1005D,1006C,1008N,1009P,1010P,1011F,1015S,1016R,1020K,1022P,1023L,1026G,1027L,1031K,1037D,1039Y,1040G,1043S,1044F,1045K,1046S,1047D,1050S,1051G,1052H,1053D,1056I,1057R,1058S,1059D,1060V,1061S,1062D,1063I,1065T,1066H,1067T,1068V,1069T,1070Y,1071G,1073I,1074E,1075G,1076N,1078A,1079K,1080R,1081R,1084Q,1085Y,1086K,1087D,1088S,1089L,1090K,1092R,1093P,1094A,1095S,1096A,1098S,1099R,1100R,1101E,1102F,1103D,1104E,1105I,1111R,1112R,1113P,1116S,1117P,1120K,1126K,1129L,1130R,1131D,1132F,1133Y,1137F,1138R,1141E,1144P,1147E,1152T,1157E,1158R,1161D,1162F,1163K,1165D,1170G,1176R,1179I,1180K,1182G,1195A,1198E,1199K,1200N,1203N,1210S,1217S,1219P,1224N,1232Q,1233N,1234S,1236R,1241R,1243E,1244A,1245C,1246K,1247K,1251L,1252Y,1253D,1255S,1257D,1260L,1263L,1264D,1266P,1268A,1271A,1288S,1289K,1295R,1298L,1299R,1300R,1301Q,1302H,1303S,1304Y,1306T,1308V,1309D,1311Q,1312K,1313E,1314E,1317L,1319P,1320R,1321S,1322V,1324L,1325K,1327K,1329R,1332D,1333G,1334S,1336Y,1338H,1339M,1341E,1342M,1344A,1349F,1353K,1358T,1360G,1368G,1370G,1372M,1376S,1384Q,1386P,1389P,1392G,1393D,1394D,1399H,1400G,1404Y,1441R,1443Q,1444K,1463R,1470N,1471G,1473V,1474Y,1477L,1479S,1480I,1481E,1482S,1483D | 14L,36G,37I,41L,47E,50I,61H,63S,69E,76T,78P,95Q,99F,102D,103T,106E,109A,113D,116S,117A,119T,125G,130S,144F,145Q,146F,147G,148P,150I,154A,156V,159N,163E,164Y,169F,173T,174T,175Y,177P,178G,201E,205L,208S,212G,214S,218N,219Q,222K,223L,224Q,225S,228I,229L,230L,233T,236E,247V,248G,250T,255T,259P,264G,270P,273F,274P,276G,280V,281S,283D,284E,287Y,291A,292R,293V,295D,296G,299I,303A,304A,305S,318K,319S,321C,327K,338Y,340I,341N,343T,344F,345E,346G,347R,348N,349L,350S,352S,353E,355G,359H,360P,361K,362L,363V,364I,365I,367L,368N,371R,376V,388Y,389Y,393R,395C,401Q,405H,410T,413E,414A,415P,418I,420E,423D,424P,430M,431R,433T,434V,435P,436C,438K,440I,444N,446T,449E,454K,456C,458K,459G,460F,461C,462I,469S,474F,477D,478L,487G,492G,496G,498I,501V,504K,508M,509A,512S,515I,517E,519R,520S,522V,523V,525F,526S,527V,528P,532T,535S,536V,538V,539S,541S,542N,545V,553P,555S,558V,561M,563F,564V,571A,573A,574V,575F,577F,584G,585Y,588C,591D,597G,598P,602I,603G,604K,605A,609L,613V,614F,615N,617S,618V,619P,620V,622N,625G,627T,629K,632V,633S,638F,640V,641I,642F,649N,652A,656Q,658E,659Y,665G,666L,668D,669K,671F,672Q,674P,675N,677F,679P,680P,681F,682R,683F,684G,685T,686V,687P,688N,689G,690S,691T,692E,693R,694N,695I,696R,697N,699Y,703H,705Y,710N,711Q,713G,714V,716D,717A,718L,720S,722K,724G,725K,726L,727D,728A,729F,731Y,732D,733A,734A,735V,737N,740A,743D,746C,751I,753S,754G,757F,758A,760T,761G,765A,767Q,768K,769D,770S,773K,775Q,777D,778L,779A,780I,781L,783L,784F,786D,787G,791E,794A,795L,798T,801C,802H,803N,806N,808V,814D,819A,820G,821V,823Y,826G,827A,839E,840H,842F,843Y,847R,850F,852G,854C,855S,856G,858P,859G,866R,867G,871C,872I,873H,874G,875V,877I,878E,879E,883V,885N,886S,887P,888T,889A,894T,895H,897N,898I,900R,901L,902L,903R,904T,906K,909A,912S,914V,915N,916G,917S,918P,919Q,920S,921A,922L,925I,926R,928E,929S,930S,931V,933D,934I,936E,937H,938R,940S,941F,942T,943H,944S,947K,948S,952P,957N,958L,960S,961D,962Y,964S,966V,967E,969T,970F,971G,972N,973L,979N,982Q,983D,984H,985Y,987H,989H,991P,992H,993S,995G,1002G,1004Y,1005D,1009P,1010P,1011F,1015S,1016R,1022P,1023L,1027L,1031K,1037D,1039Y,1040G,1043S,1044F,1045K,1046S,1051G,1052H,1053D,1056I,1057R,1058S,1059D,1060V,1061S,1062D,1063I,1065T,1066H,1067T,1068V,1069T,1070Y,1071G,1074E,1075G,1076N,1079K,1080R,1081R,1084Q,1085Y,1087D,1088S,1090K,1092R,1093P,1094A,1095S,1096A,1099R,1100R,1101E,1103D,1104E,1105I,1111R,1112R,1113P,1120K,1126K,1129L,1130R,1131D,1132F,1133Y,1137F,1138R,1141E,1144P,1147E,1157E,1165D,1170G,1176R,1182G,1195A,1198E,1199K,1200N,1203N,1217S,1234S,1241R,1243E,1244A,1245C,1246K,1247K,1251L,1252Y,1255S,1257D,1271A,1289K,1298L,1299R,1300R,1301Q,1302H,1303S,1304Y,1306T,1308V,1311Q,1313E,1314E,1319P,1320R,1321S,1322V,1324L,1325K,1327K,1332D,1333G,1334S,1336Y,1341E,1344A,1368G,1376S,1384Q,1386P,1389P,1392G,1393D,1394D,1399H,1400G,1444K,1470N,1471G,1473V,1477L,1479S,1481E,1482S,1483D | |
| 2C | 28Q,33A,37S,47R,49R,50L,51T,53Q,59P,64P,66T,67V,69V,70N,73N,74P,75S,77L,78L,79T,82C,85L,92G,94V,95F,96E,97D,98N,101T,103A,105A,106Q,108L,109D,115T,118P,119I,120L,121S,123S,124G,125G,126S,127A,128V,131T,132P,135P,136G,137S,139F,140L,141Q,143G,144V,149Q,155K,158E,159E,160Y,165F,166A,169T,170S,173P,180E,186A,187D,190H,197D,198V,200T,201L,209R,213Q,214R,215L,216L,220D,221A,223V,226A,228C,229S,230R,231E,232E,233A,234E,235V,236L,237F,240A,244G,245L,247G,248P,249G,253L,255P,256N,258A,260G,263D,266P,267A,270P,272G,273L,274I,275S,276V,278T,279E,283L,285L,286R,287Q,288K,289V,290R,291D,292G,293V,294A,295I,298L,299G,303Y,308G,310L,311P,316D,334H,335L,337N,338V,339T,341E,343R,344D,346S,347F,351G,352Y,353L,356P,361I,364N,365R,366H,367R,373G,375W,378G,383K,384Y,385P,388P,389R,390Y,396P,398V,399D,401R,402H,404T,405V,407T,409E,410E,411R,412P,413F,415I,416V,420D,421P,423T,424G,426C,427V,430T,431V,434R,435R,440T,441F,444G,452L,453C,454C,455K,459I,471F,473Y,475L,480N,481G,482K,483H,485K,487V,488R,489G,492N,493G,495I,496G,502R,503A,508G,510L,513N,514E,515E,517S,519I,521D,522F,523S,524V,525P,526F,529T,530G,533V,537R,538S,539N,541T,543S,544P,545S,547F,548L,550P,552S,553P,554A,557V,558M,560F,561V,565T,566V,568A,570T,571V,572F,574F,575E,578S,579P,580V,585N,592S,593G,594G,595P,597F,598T,599I,602S,608A,611F,614S,615V,618E,620P,621R,623T,625S,626K,628M,629V,633A,635F,636A,641A,657I,659T,661S,662G,665D,666K,668F,671P,675Y,676P,678F,680F,684P,689E,690R,692I,693R,695N,705K,708Q,710S,713D,714A,719K,722K,724D,726F,728Y,729D,730A,731A,733L,734N,737A,738G,739K,741E,744K,746V,751G,752K,754F,755A,757T,761I,762A,765K,766D,770K,773I,775L,777L,778L,780F,782G,783D,784G,786T,788K,791T,792V,794L,796G,800N,801E,803N,806M,812I,815M,816A,818V,819F,832V,833F,834A,837H,842K,844R,845H,846S,849N,850S,853L,860S,862G,865S,866C,869G,870V,882P,883D,892S,894L,895K,898Q,912S,913L,917T,920I,963P,1037R,1050E | 33A,47R,49R,50L,51T,53Q,59P,64P,66T,67V,69V,70N,73N,74P,75S,77L,79T,82C,85L,92G,94V,95F,96E,97D,101T,103A,105A,106Q,108L,109D,115T,118P,123S,124G,125G,126S,127A,128V,131T,135P,136G,137S,139F,140L,141Q,143G,149Q,155K,158E,159E,160Y,165F,166A,169T,170S,173P,180E,186A,187D,190H,215L,216L,220D,221A,223V,226A,228C,229S,230R,231E,232E,233A,235V,237F,240A,244G,245L,247G,248P,249G,253L,255P,256N,258A,260G,263D,266P,267A,270P,272G,273L,275S,278T,283L,285L,286R,288K,289V,290R,291D,292G,293V,294A,295I,299G,303Y,308G,311P,316D,334H,335L,337N,338V,339T,341E,343R,344D,346S,347F,351G,353L,356P,365R,367R,373G,375W,378G,383K,384Y,385P,388P,389R,390Y,396P,398V,399D,401R,402H,404T,405V,407T,409E,410E,411R,412P,413F,415I,416V,420D,421P,423T,424G,426C,430T,434R,435R,444G,452L,454C,455K,459I,471F,473Y,475L,480N,481G,482K,483H,487V,489G,492N,493G,495I,502R,503A,508G,513N,515E,519I,522F,523S,524V,526F,529T,530G,533V,537R,538S,539N,541T,543S,544P,547F,548L,550P,553P,554A,560F,561V,565T,566V,568A,571V,574F,575E,578S,580V,585N,592S,593G,594G,595P,598T,602S,614S,615V,618E,620P,625S,629V,633A,635F,641A,661S,662G,668F,675Y,678F,680F,690R,692I,693R,710S,719K,724D,729D,730A,731A,733L,734N,737A,738G,739K,741E,746V,752K,754F,755A,757T,761I,765K,770K,773I,775L,777L,778L,780F,783D,784G,788K,791T,800N,801E,803N,806M,812I,816A,818V,832V,833F,842K,844R,845H,846S,849N,850S,853L,860S,865S,869G,882P,892S,894L,912S,917T,920I,963P | |
| 2D | 41P,84V,92N,94S,95D,96P,102Q,103L,104C,108S,114G,115V,116V,118E,119D,120D,124P,126V,127A,130L,131D,132F,134S,135A,137T,140P,142V,146G,151V,153T,154P,156E,158G,161F,162L,164L,166S,168T,171Q,172L,175I,176F,177E,178V,179L,180E,182Y,183D,185T,187F,188V,192T,195P,197H,199A,200F,205E,208T,209D,210G,211S,213V,217H,219G,224D,234A,235Q,241A,243I,244R,245L,246L,251E,252E,253A,254E,256V,257F,259A,260A,261E,262E,266T,270Y,273F,274M,276G,277P,279L,280A,305F,306A,308R,309S,311G,313R,314D,315D,316L,318R,319R,321A,323G,325A,330G,331A,333A,334L,336R,339G,344L,347D,348C,351Q,363Y,367I,368T,369W,374Y,376F,377N,380G,381F,384N,385P,388V,389V,396R,399E,401V,402G,405E,406Q,409L,410R,412K,414P,417S,418R,419Y,420G,423L,425P,427D,428D,432L,433T,434V,435A,436T,437L,439E,440R,441P,442F,443V,444I,446E,448A,450P,457R,459S,460V,463R,465Q,467N,475D,482R,485K,486G,489I,492L,494R,496A,497H,498T,499I,500G,503Y,505L,506Y,507L,510N,511G,512K,513H,514G,515K,516K,517I,518D,519G,522N,525I,526G,532R,533A,534D,542I,543N,544E,549I,551D,556F,557V,559T,564M,567R,571T,572V,573S,574P,575S,577F,580P,582S,583P,584A,590F,595T,596V,597V,598A,599V,600T,603I,604F,605E,608S,609P,610V,611G,612Y,616L,620K,622P,623G,624G,625S,627F,628T,629I,630G,631K,632S,635L,636L,638A,640V,641F,642N,644S,645V,646P,649N,650P,651R,652G,654T,665F,666A,669F,670L,675A,678A,685E,689T,691S,693L,695D,696R,698F,700R,701P,704Q,705Y,706P,707P,710F,711G,712T,713V,715N,716G,718T,720K,721N,722I,726Y,730H,735R,742E,748L,751G,752K,754D,756F,757I,758Y,759D,760A,761A,762V,763L,765Y,768R,769K,770D,771E,773C,774K,775L,778I,780S,782K,783V,784F,786T,787T,788G,789Y,790G,793L,795K,797S,800K,801R,802P,803I,806A,807L,813D,815E,816I,817E,819L,821R,822L,824L,825S,829H,830N,831D,833I,841D,842I,846A,847G,848V,852L,854V,858L,860L,866E,868L,870Y,872R,873L,874R,876C,886L,887L,891R,892G,895S,900E,901A,907K,914P,918P,922A,938A,951P,963R,967P,968I,972G,985R,1016P,1048L,1094P,1095P,1154S,1155V,1156D,1192P,1217G,1252R | 92N,95D,96P,102Q,104C,108S,114G,115V,116V,118E,119D,127A,132F,134S,135A,137T,140P,146G,151V,154P,156E,158G,162L,164L,168T,171Q,172L,175I,176F,177E,178V,180E,182Y,183D,185T,187F,188V,192T,195P,197H,199A,200F,205E,208T,209D,210G,224D,234A,243I,244R,245L,251E,252E,253A,254E,257F,259A,260A,262E,266T,273F,274M,276G,277P,305F,309S,311G,314D,315D,316L,318R,319R,323G,325A,330G,331A,333A,336R,339G,344L,347D,367I,368T,376F,380G,384N,385P,389V,399E,401V,402G,405E,410R,412K,414P,417S,418R,420G,423L,425P,427D,428D,432L,433T,434V,435A,436T,437L,439E,440R,441P,442F,443V,444I,450P,457R,459S,460V,463R,465Q,467N,482R,485K,486G,489I,492L,494R,496A,498T,500G,503Y,505L,506Y,507L,510N,511G,512K,513H,514G,515K,517I,518D,519G,522N,526G,532R,533A,534D,542I,544E,556F,557V,559T,564M,571T,572V,574P,575S,577F,580P,582S,583P,584A,590F,595T,596V,597V,598A,599V,600T,603I,604F,605E,608S,609P,610V,611G,612Y,616L,620K,623G,624G,625S,627F,628T,629I,630G,631K,632S,636L,638A,640V,641F,642N,644S,645V,646P,649N,650P,651R,652G,654T,665F,666A,669F,675A,678A,685E,689T,691S,693L,695D,696R,698F,700R,701P,705Y,706P,707P,710F,711G,712T,713V,715N,716G,718T,721N,722I,726Y,730H,735R,742E,748L,751G,752K,754D,756F,757I,758Y,759D,760A,761A,762V,765Y,769K,770D,771E,773C,774K,775L,778I,780S,782K,783V,784F,786T,787T,788G,789Y,790G,793L,795K,797S,800K,801R,802P,806A,807L,813D,815E,816I,819L,821R,822L,824L,825S,829H,830N,831D,833I,841D,842I,846A,847G,848V,852L,854V,858L,860L,866E,868L,870Y,874R,876C,892G,895S,900E,901A,922A,938A,951P,963R,967P,968I,972G,1016P,1094P,1095P,1154S,1155V,1156D | |

Two hundred and twenty-four sites were investigated based on posterior probability (Qk) within GRIN2s. Sites with Qk>0.67 or Qk>0.9 were listed relative to human GRIN2A protein sequence. Sites located at N-terminal transmembrane regions M1, M3 or re-entrant loop (M2) were colored red, green or pink, respectively. Sites under negative selection (*P*<0.1) or strong negative selection (*P*<0.05) were listed relative to human GRIN2s protein sequences, respectively.
